# The essential role of DSMBs in ensuring the ethics of global vaccine trials to address COVID-19

**DOI:** 10.1093/cid/ciab239

**Published:** 2021-03-24

**Authors:** Lisa Eckstein, Annette Rid, Dorcas Kamuya, Seema K Shah

**Affiliations:** 1 School of Law, University of Tasmania, Australia; 2 Clinical Center Department of Bioethics & Division of AIDS, National Institutes of Health, USA; 3 Health Systems and Research Ethics (HSRE) Department, Kenya Medical Research Institute (KEMRI)-Wellcome Trust Research Programme, Kenya; 4 Department of Pediatrics, Northwestern Feinberg School of Medicine (USA); Mary Ann & J. Milburn Smith Child Health Outcomes, Research, and Evaluation (SCHORE) Center; Stanley Manne Children’s Research Institute; Lurie Children’s Hospital (USA)

**Keywords:** SARS-CoV-2, COVID-19 Vaccines, Clinical Trials Data Monitoring Committees, Standard of Care, Ethics, Research

## Abstract

COVID-19 vaccines are being developed and implemented with unprecedented speed. Accordingly, trials considered ethical at their inception may quickly become concerning. We provide recommendations for Data and Safety Monitoring Boards (DSMBs) on monitoring the ethical acceptability of COVID-19 vaccine trials, focusing on placebo-controlled trials in low- and middle-income countries.

## Introduction

Over seventy vaccine candidates to protect against COVID-19 are currently being tested in humans. At the time of writing, three have received emergency use authorization (EUA) in the United States, and ten vaccines have been approved or authorized in different countries.[1] Additional authorisations and approvals are likely and will help accelerate the response to the global COVID-19 pandemic, alongside scaling up of manufacturing and distribution.

Despite these developments, more COVID-19 vaccine research is needed. EUAs require more limited data than full approvals, and some questions remain about currently authorized vaccines, including their long-term safety and efficacy in different populations and whether they prevent viral transmission. The first vaccines to be authorized were developed by Pfizer/BioNTech and Moderna and use messenger RNA technology (mRNA) technology. These mRNA vaccines appear highly efficacious but will face implementation challenges in low- and middle-income countries (LMICs); they are expensive, require two shots, and might need to be stored at ultra-cold temperatures. AstraZeneca's vaccine is lower-cost and stored at ordinary refrigerated temperatures; however, it seems less efficacious and warrants further study because of dosing discrepancies in the Phase III trial.[2] Johnson and Johnson/Janssen's vaccine was most recently authorized. It requires only one dose and can be stored in standard refrigerators (between 2 and 8 degrees Celsius), making it much easier to distribute globally, but supply will be limited. Although the two mRNA vaccines appear to have the highest efficacy, their protection against newer variants of the novel coronavirus is unknown, some of which appear more likely to evade protection. More testing will therefore be needed for most of the existing vaccines against emerging variants.

Yet future COVID-19 vaccine trials in LMICs are likely to face complex ethical questions about study design: the acceptability of placebo controls [3–5] will hinge on the limitations of existing data, local vaccine availability, and community views—all of which could change rapidly. In accordance with pre-specified monitoring plans, DSMBs provide recommendations to trial sponsors and/or trial steering committees based on this emerging data. Data and Safety Monitoring Boards (DSMBs)—constituted by research sponsors to independently examine ongoing trial data—typically are responsible for monitoring the safety and acceptability of clinical trials as data emerges. In this commentary, we identify challenges that DSMBs may face in evaluating the ethics of ongoing COVID-19 vaccine trials, including in LMICs, and provide recommendations for addressing them.

## Guidance on Placebo-Controlled Trials in LMICs

Placebo-controlled trials are generally considered the 'gold standard' for evaluating novel interventions. However they have been controversial when a proven, effective standard of care or prevention exists.[6] After much debate, placebo-controlled trials are now considered ethically acceptable, even when an established, effective intervention exists, under the following conditions:

1. Responsiveness of the research to local health priorities; and
2. A compelling scientific rationale for using placebo; and
3. A level of risk to participants in the placebo group that is justified by the social value of the research.[3,6–8]

First, research is responsive if the knowledge gained from a trial could improve a country's local standard of care.[9] To determine if placebo use is acceptable, it is not enough to look to the *de facto* standard of care in a country, or what *is* currently being provided. Instead, acceptability depends on the *de jure* standard of care—what *should* be provided based on a reasonable approach to allocating scarce resources locally.[10] Imagine there is a single-dose, highly efficacious vaccine for COVID-19 that requires no cold chain and is widely available at low cost through the Global Alliance for Vaccines and Immunizations (GAVI). Such a vaccine would be the *de jure* standard of care, even if

*de facto* some LMICs are slow to ensure widespread access to such a product. By contrast, at the time of writing, the Pfizer/BioNTech and Moderna vaccines for COVID-19 are not yet reasonably available in LMICs, given limited supplies, high costs, ultra-cold chain storage requirements and a two-dose regimen that is challenging to implement. The AstraZeneca vaccine will likely be more accessible, but it still requires cold storage and may require two doses.[2] Johnson & Johnson's Janssen single dose vaccine is stable at 36-46 degrees Fahrenheit, enabling "distribution through standard vaccine channels", though authorization and scale-up of production will take time.[11] COVID-19 vaccination is not yet the *de jure* standard of care in LMICs; placebo-controlled vaccine trials could therefore be responsive to local health priorities if they can help improve this standard of care by testing vaccines that are easier to implement in LMICs.

Additionally, to ensure research is responsive, sponsors and researchers should work with local governments and other stakeholders to develop credible pathways for making safe and effective vaccines available to the population. This involves collecting data relevant for local licensure, setting a fair price, making necessary funds and infrastructure available for roll-out, and establishing mechanisms for conducting safety surveillance of administered vaccines. Since an investigational vaccine might not prove safe and/or effective, sponsors and researchers should ensure research benefits are shared fairly overall by providing additional benefits to local communities, such as investing in local health infrastructure. Which benefits are shared should be determined in consultation with the community.[7]

Second, to have a compelling scientific rationale, a placebo-controlled design should have significant methodological advantages over an active-controlled design for addressing the research question. In COVID-19 vaccine trials, such advantages exist when data on an established vaccine may not generalize to the local population.[5] A placebo-controlled design can also have significant advantages when the vaccine being tested is responsive to local health needs and expected to offer robust protection, but the level of protection is likely lower than for established vaccines.[6] In this scenario, the relevant question for local vaccine licensure and use is safety and efficacy of the vaccine compared to no vaccination—not when compared with other vaccines authorized elsewhere.

Third, the level of risk to participants in the placebo group must be justified by the social value of the research. Because the placebo itself carries few risks, the key issue is whether participants are denied a vaccine to which they are otherwise entitled (i.e. the *de jure* standard of care). If so, their lack of protection is the relevant research-related risk that requires ethical scrutiny. There is agreement that the risks from receiving placebo should be minimized, for example, through promptly providing care for participants who become infected. However, once minimized, what level of risk is justifiable remains contested. The Council for International Organizations of the Medical Sciences indicates participants should be exposed to no more than a minor increase over minimal risk .[7] Yet this approach is more restrictive than for research interventions other than placebo, since competent adults can normally consent to higher risks provided the risks are justified by the social value of the research. Accordingly, a World Health Organization panel applied this standard approach to risk/benefit evaluations, determining that the risks of receiving placebo should be justified by the trial's social value.[8] Consistent with the first two conditions of acceptable placebo use that we mention above, social value in this case depends on the value of the knowledge produced by the research for improving health, including whether the research has a compelling scientific rationale and is responsive to local health priorities.[6,8]

## Implementing ethical guidance for COVID-19 vaccine trials

There is an emerging agreement based on these criteria that placebo-controlled COVID-19 vaccine trials could be ethically acceptable depending on the circumstances.[3–5] The scientific goal of these trials would be to demonstrate sufficient efficacy and safety to warrant approval, *even if* the candidate vaccine was somewhat less effective than those already authorized. Although a non-inferiority design could theoretically produce needed data,[12] comparing a candidate vaccine to an already authorized one poses logistical, methodological, and interpretative challenges.[13] A placebo-controlled trial would therefore be preferable for scientific reasons—and as long as the *de jure* standard of care is no vaccination, participants in the placebo arm would be exposed to limited research-related risks (e.g., from the placebo injection or study-related blood draws).[14] However, the justification for such a placebo-controlled trial could diminish rapidly if already-authorized or new vaccines become more accessible or implementable. For example, with the authorization of the Johnson & Johnson vaccine, placebo use might not be justified if manufacturing capacity is rapidly scaled up to meet global need.[15]

Despite growing consensus on the relevant criteria, making these judgments in practice is difficult. Before trials start, local research ethics committees (RECs) must establish that criteria for ethical acceptability are met. National policy-makers, in consultation with communities and international bodies, can provide RECs with guidance on a country's standard of care regarding COVID-19 vaccination.[16] RECs should also ensure the informed consent process clearly explains the rationale for placebo controls, when they will be unblinded, and when they might access an authorized vaccine.[17]

Once trials begin, however, IRBs/RECs lack the contextualised information and expertise to monitor interim data.[18] Based on concerns about scientific integrity, including guarding against sponsor or researcher bias, DSMB members are the only individuals provided with access to unblinded interim data.[17] DSMBs therefore have the sole capacity to monitor this interim data for safety, efficacy, and/or futility in order to assess a trial's ongoing acceptability. The scope of DSMB review—including the criteria on which DSMBs can or should make recommendations to trial sponsors and/or trial steering committees whether trials should be continued, stopped, or modified—depends on trial-specific monitoring plans. Commonly, these plans include statistical "stopping boundaries"; however, it is widely accepted that these operate as guidelines rather than rules, and that the DSMB role extends to a holistic assessment of a trial's risks and benefits to participants and society.[19] Accordingly, DSMB recommendations, and the plans upon which they are based, can require complex value judgments.

When overseeing placebo-controlled COVID-19 vaccine trials in LMICs, DSMBs will face the challenge of evaluating interim data with a rapidly evolving local standard of care. With fast-paced scientific progress—and new social, economic and political developments—already-authorized vaccines that are not widely accessible at the beginning of a trial could become the *de jure* standard of care during the research, making initial REC judgments outdated. To ensure the ongoing ethical acceptability of placebo-controlled COVID-19 vaccine trials in LMICs, sponsors and/or steering committees with decision-making responsibility for an ongoing trial will need to select DSMB members who have no conflicts of interest, and draft a charter and monitoring plan that allows for the DSMB to make rigorous independent judgments. DSMBs will then have to re-evaluate participant risks and benefits and continued feasibility in accordance with information about the emerging *de jure* standard of care for trial sites. However, DSMBs are likely to face two significant challenges in carrying out this charge.

## Challenges for DSMBs

First, not all DSMBs have the expertise to address ethical questions,[20,21] in line with broader gaps in training for DSMB members. [22] While DSMBs have specialized statistical and clinical expertise, there are few other membership requirements. World Health Organization operational guidelines indicate ethicists are sometimes required for "certain" (unspecified) studies.[23] There is also no consensus on including members with ties to the local community. Unfortunately, the scope of the problem is difficult to determine as DSMB membership is often kept confidential to protect scientific integrity.[24]

Second, DSMBs lack substantive ethical guidance for the value judgments they are often required to make. Trial protocols typically reflect a procedural and technical approach to monitoring. These plans offer guidelines, not rules. While the U.S. FDA indicates that DSMBs need not recommend termination when boundaries are crossed, “since other aspects of the interim data may complicate the issue,” it then merely advises sponsors to “direct the [DSMB] to exercise its own judgment in such circumstances.”[25] Even in the high-stakes context of the COVID-19 pandemic, protocols for the Moderna and Pfizer/BioNTech vaccine trials included limited guidance.[26,27] For example, the Moderna protocol provided statistical stopping boundaries but merely required the DSMB to record and communicate to the Sponsor any reason for disregarding a boundary. The application of stopping boundaries could be further complicated by potential gaps in background disease surveillance, particularly in LMICs,[28] which can help interpret emerging trial data and assess the safety and efficacy of vaccine roll-out.

Without a clearer framework for following or deviating from statistical analysis plans, it is difficult for sponsors, trial steering committees, and others to evaluate the wide range of DSMB recommendations. For example, in the Adaptive COVID-19 Treatment Trial, the DSMB recommended early termination of the placebo arm based on interim data showing that participants treated with remdesivir recovered faster than those receiving placebo.[29] Although this decision promoted the interests of participants in the placebo arm in receiving a seemingly efficacious therapy when little was known about COVID-19, some questioned the DSMB’s recommendation, given the social value of collecting additional data on remdesivir’s potential mortality benefits.[29] Indeed, the SOLIDARITY trials’ results found no benefit from remdesivir.[30] In other trials, DSMBs have recommended continuing placebo arms even after interim data crossed a prespecified stopping boundary in order to gather additional data, for example, on adverse events, prioritizing the social value of the research for future patients.[31]

## The path forward

To address these challenges in the context of COVID-19 vaccine trials, sponsors, trial steering committees and others with responsibility for constituting DSMBs should select members and draft monitoring plans that will support the independent and value-laden nature of their decision-making. Once DSMBs do make recommendations, sponsors and trial steering committees should be prepared to act on these recommendations,[32] or, in the event of a reasonable disagreement, to engage in a robust process for resolution.[33]

As a starting point, including ethicists and experts on the DSMB with strong ties to local communities may help open important channels of communication and sensitize the DSMB to relevant ethical issues and local circumstances. For example, local experts may be best able to determine whether vaccine supply is unjustly allocated in host countries or communities, resulting in a gap between the *de jure* and *de facto* standards of care. While previous capacity building efforts in LMICs have resulted in well-established clinical trial infrastructure and research ethicists at many centers,[34] identifying and training qualified DSMB members can be challenging. Online training materials about the role and operation of DSMBs are becoming available.[22] However, more materials would help, including specific guidance on the ethical issues faced by DSMBs. DSMBs should also have access to local independent consultants with health policy expertise.[23]

Second, protocols and statistical analysis plans should expressly recognize the value-laden nature of trial monitoring, including the need to evaluate continued placebo use in light of the *de jure* standard of care. Stopping boundaries could include potential changes in whether some or all participants should be receiving access to vaccines, alongside safety and efficacy data. Alternatively, factors for deviating from the statistical analysis plan could be explicitly delineated. Second, to allow this assessment, sponsors—ideally in partnership with local health authorities—should provide reports to DSMBs about such availability. Finally, greater clarity is needed on the relationship between the respective roles of DSMBs and RECs. In some cases, rather than merely sharing DSMB reports with RECs, greater collaboration between DSMBs and RECs may be helpful.[35–37] This could include prespecifying circumstances where a DSMB should brief one or more RECs on emerging trial data in order for the REC to make a determination about a trial's ongoing ethical acceptability.[37] Another option could be greater integration of the two bodies through the inclusion of an REC member on a DSMB.

**Acknowledgements**

The authors would like to thank Kathryn M. Edwards, Mainga Hamaluba, and David Wendler for helpful comments on a previous version, as well as two anonymous reviewers. This work was supported in part by the Clinical Center Department of Bioethics, which is in the Intramural Program of the National Institutes of Health. DK is funded by the Wellcome Trust [205419/Z/16/Z]. For the purpose of Open Access, the author has applied a CC-BY public copyright licence to any author accepted manuscript version arising from this submission.

**Disclaimer:** The views expressed here are those of the author and do not necessarily reflect the policies of the National Institutes of Health or the U.S. Department of Health and Human Services. The manuscript is published with support from the Director, Kenya Medical Research Institute.

**Conflicts of Interest:** LE holds shares in Johnson & Johnson and a biotechnology index fund, whose holdings include Moderna, Novartis, BioNTech and Novovax. LE is chair of the Tasmania Health and Medical Human Research Ethics Committee and serves on an Observational Safety Monitoring Board for a National Heart, Lung, and Blood Institute study. SKS serves on a National Institute of Allergy and Infectious Diseases DSMB. DK reports research fellowship grants from Wellcome Trust.  All other authors have no potential conflicts.

**Table 1: Summary of Needed Attributes of DSMBs for Global COVID-19 Vaccine Trials**

| | |
|---|---|
| DSMB membership | Where possible, identifying and training locally-linked ethicists and/or community representatives. Independence of DSMB members from sponsor company. |
| Statistical analysis plan (SAP) | SAPs, including stopping boundaries, should explicitly incorporate ethical dimensions of monitoring, and/or factors for deviating from original plan |
| Provision of relevant information | Reports made to DSMB on local circumstances, such as local vaccine availability, distribution to date, and timeline for wider distribution to inform decision-making |
| Wider consultation in decision-making | Consultative decision-making models in certain circumstances, including with local RECs |
